# Determinants of Antibiotic Resistance and Virulence Factors in the Genome of *Escherichia coli* APEC 36 Strain Isolated from a Broiler Chicken with Generalized Colibacillosis

**DOI:** 10.3390/antibiotics13100945

**Published:** 2024-10-09

**Authors:** Dmitry S. Karpov, Elizaveta M. Kazakova, Maxim A. Kovalev, Mikhail S. Shumkov, Tomiris Kusainova, Irina A. Tarasova, Pamila J. Osipova, Svetlana V. Poddubko, Vladimir A. Mitkevich, Marina V. Kuznetsova, Anna V. Goncharenko

**Affiliations:** 1 Center for Precision Genome Editing and Genetic Technologies for Biomedicine, Engelhardt Institute of Molecular Biology, Russian Academy of Sciences, 119991 Moscow, Russia; aleom@yandex.ru (D.S.K.); kovalev_maksim_2002@mail.ru (M.A.K.); mitkevich@gmail.com (V.A.M.); 2 V.L. Talrose Institute for Energy Problems of Chemical Physics, N.N. Semenov Federal Research Center for Chemical Physics, Russian Academy of Sciences, 119334 Moscow, Russia; kazakovaem@gmail.com (E.M.K.); kusainova7531@gmail.com (T.K.); iatarasova@yandex.ru (I.A.T.); 3 Bach Institute of Biochemistry, Fundamentals of Biotechnology Federal Research Center, Russian Academy of Sciences, 119071 Moscow, Russia; shumkovm@gmail.com; 4 Institute of Biomedical Problems of Russian Academy of Sciences, 123007 Moscow, Russia; osipova.pamila@yandex.ru (P.J.O.); poddubko@imbp.ru (S.V.P.); 5 Perm Federal Research Centre, Institute of Ecology and Genetics of Microorganisms, Ural Branch Russian Academy of Sciences, 614081 Perm, Russia; mar@iegm.ru

**Keywords:** *Escherichia coli*, colibacillosis, virulence-associated genes, antibiotic resistance

## Abstract

**Objective:** Multidrug-resistant, highly pathogenic *Escherichia coli* strains are the primary causative agents of intestinal and extraintestinal human diseases. The extensive utilization of antibiotics for farm animals has been identified as a contributing factor to the emergence and dissemination of *E. coli* strains that exhibit multidrug resistance and possess high pathogenic potential. Consequently, a significant research objective is to examine the genetic diversity of pathogenic *E. coli* strains and to identify those that may pose a threat to human health. **Methods:** In this study, we present the results of genome sequencing and analysis, as well as the physiological characterization of *E. coli* strain APEC 36, which was isolated from the liver of a broiler chicken with generalized colibacillosis. **Results:** We found that APEC 36 possess a number of mechanisms of antibiotic resistance, including antibiotic efflux, antibiotic inactivation, and antibiotic target alteration/replacement/protection. The most widely represented group among these mechanisms was that of antibiotic efflux. This finding is consistent with the strain’s documented resistance to multiple antibiotics. APEC 36 has an extremely rare variant of the beta-lactamase CTX-M-169. Notwithstanding the multitude of systems for interfering with foreign DNA present in the strain, seven plasmids have been identified, three of which may possess novel replication origins. Additionally, *qnrS1*, which confers resistance to fluoroquinolones, was found to be encoded in the genome rather than in the plasmid. This suggests that the determinants of antibiotic resistance may be captured in the genome and stably transmitted from generation to generation. **Conclusions:** The APEC 36 strain has genes for toxins, adhesins, protectins, and an iron uptake system. The obtained set of genetic and physiological characteristics allowed us to assume that this strain has a high pathogenic potential for humans.

## 1. Introduction

The uncontrolled use of antibiotics in medicine and agriculture over an extended period has resulted in the proliferation of antimicrobial-resistant (AMR) strains of microorganisms. Consequently, the World Health Organization (WHO) has indicated that drug-resistant microorganisms are responsible for the deaths of more than 1.2 million individuals annually, with this figure projected to reach 10 million by 2050 [1,2]. Furthermore, the World Bank projects that AMR could result in an additional USD 1 trillion in healthcare expenditures by 2050. At present, the most common AMR bacterial causative agent is *Escherichia coli*, accounting for 43% of cases [2]. The WHO has identified antibiotic resistance as one of the most significant public health threats. Given that farm animals are considered a primary source of human pathogens including *E. coli*, it is essential to monitor the actual pathogens of farm animals for the presence of antibiotic resistance and virulence genes.

Commensal non-pathogenic *E. coli* are normal microbiota of the gastrointestinal tract of humans and animals. However, some representatives of this species of bacteria have the potential to cause diseases of the digestive system and generalized systemic infections [3]. Cattle are recognized as the principal reservoir for diarrheagenic *Escherichia coli* (DEC). Concurrently, poultry represents a significant source of extraintestinal pathogenic *Escherichia coli* (ExPEC) strains [4,5,6,7]. The virulence potential of *E. coli* is attributable to the presence of pathogenicity genes that encode adhesins, invasins, toxins, resistance factors to immune system, and iron uptake system proteins [8]. The intraspecific heterogeneity of this microorganism, which is due to the possibility of horizontal transfer of genetic determinants associated with virulence and antibiotic resistance, provides a high degree of plasticity and adaptability of bacteria in the changing conditions of pathogen habitats, including those in the closed circuit of agro-industrial complexes [9,10]. Consequently, hybrid strains of *E. coli* emerge, exhibiting a combination of genetic traits from diverse pathotypes of *E. coli* and a multidrug-resistant (MDR) phenotype [11].

The development of bacterial resistance to antibacterial drugs represents a significant challenge in the context of livestock production, particularly given the potential for agricultural businesses to serve as hotspots for the emergence and dissemination of antibiotic-resistant pathogens [12,13]. The transmission of antibiotic-resistant microorganisms from animals to humans and vice versa is a documented phenomenon. The dissemination of these bacteria into the environment is also facilitated by the use of organic fertilizers and farm waste products [14]. Modern genomic technologies permit the tracking of the representation of antibiotic resistance genes and the genetic “organization” of the resistome (e.g., the identification of mobile genetic elements, including plasmids, transposons, and integrons) within and between microbial populations. Furthermore, the implementation of these techniques allows for the tracking of the sources of antibiotic-resistant pathogens and the modeling of the evolutionary relationships and transmission of antimicrobial resistance determinants [15].

Therefore, the most crucial research objective at this juncture is to examine the genetic diversity of pathogenic *E. coli* strains and to ascertain those strains that are potentially pathogenic to humans. The present study is focused on the bioinformatic analysis of the genome and partial physiological characterization of an *E. coli* MDR strain isolated from the liver of a broiler chicken with generalized colibacillosis.

## 2. Results

### 2.1. Genome Sequencing-Based General Characterization and Identification of E. coli Strain APEC 36

An analysis conducted using the Quast program (version 5.2.0, [16]) which evaluates the quality of genome assembly, revealed that the results of sequencing and primary data processing yielded eight circular contigs, each exceeding1000 bp in length, with a total length of 4,979,540 bp. The length of the longest contig 5 was 4,629,530 bp, which is presumed to be the genome. The N50 and N90 values were both 4,629,530, while the L50 and L90 values were both 1. The coverage value was 135. The proportion of GC pairs was determined to be 50.74%. The data obtained indicate that the genome assembly is of a high quality. As indicated by the data from the BUSCO program BUSCO program (version 5.5.0 [17]), the completeness of the strain genome assembly, estimated from characterized genomes of the order Enterobacterales, is 98.9%. A total of 435 complete gene copies and five fragmented genes were identified from 440 gene groups. The level of contamination, as determined by the MiGA online server [18], was found to be 0.9%. Therefore, the obtained quality of the sequenced genome of strain APEC 36 is sufficient for further more detailed analysis.

The gene annotation program Prokka [19] identified 4762 protein-coding genes in the genome, of which 1009 are hypothetical and 3753 have functional annotation. Additionally, the genome contains 22 rRNA genes, one of which is duplicated, 88 tRNA genes, and one tmRNA gene. An analysis conducted using the RASTtk program [20] identified 4989 protein-coding genes (of which 591 genes are hypothetical and 4398 have functional annotation), 86 tRNA genes, and 21 rRNA genes.

As indicated in the PubMLST database [21], the ribosomal MLST profile is identical to rST 113079, which corresponds to the *E. coli* species (taxon identifier in NCBI: 1452441). The MLST scheme for the identification of strains of *E. coli* employs the analysis of seven housekeeping genes: adenylate kinase *adk*, class II fumarate hydratase *fumC*, DNA gyrase beta subunit *gyrB*, isocitrate dehydrogenase *icd*, malate dehydrogenase *mdh*, phosphoribosylaminoimidazolecarboxamidformyltransferase *pur*, and recombinase *recA* [21]. The combination of alleles observed in the strain under consideration corresponds to the ST-695 sequence type, as detailed in (Table 1).

**Figure 1:**
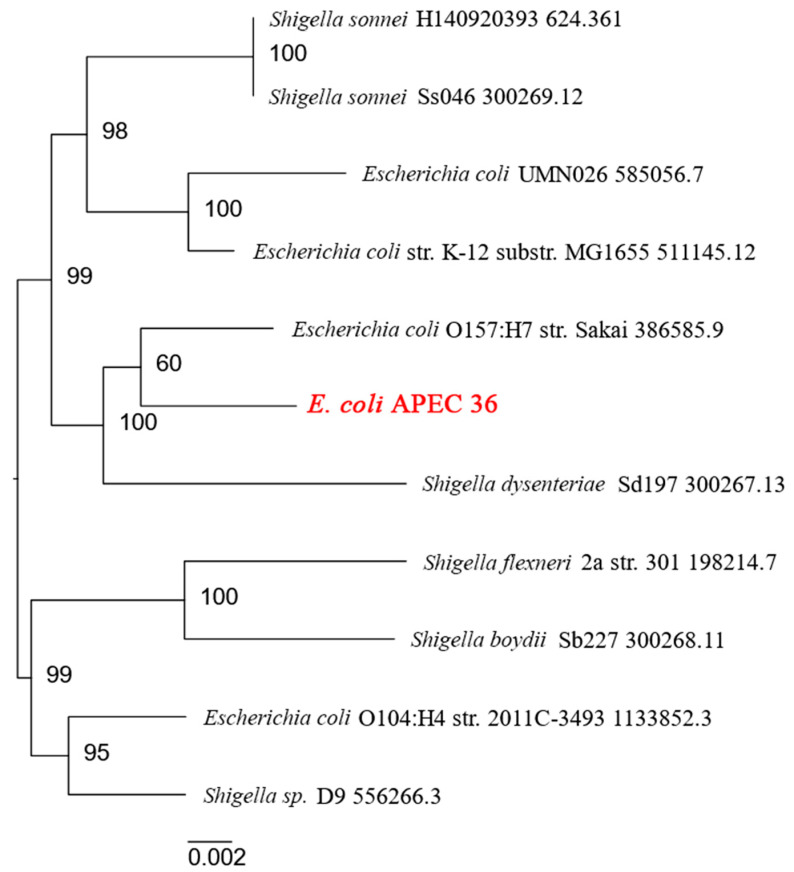
Human pathogenic strains close to *E. coli* APEC 36 according to the BV-BRC database.

A phylogenetic analysis conducted using the BV-BRC database, which stores information on human microbial pathogens [22], revealed that *E. coli* strain APEC 36 is closely related to *E. coli* strain O157:H7 Sakai [23] (Figure 1). *E. coli* O157:H7 is a significant human pathogen that causes hemorrhagic colitis and the hemolytic-uremic syndrome. It has the potential to cause severe illness and large-scale outbreaks globally [24]. These findings suggest that *E. coli* APEC 36 may also have a high pathogenic potential for humans.

PlasmidFinder 2.1 [25] was employed to identify plasmids in the APEC 36 strain. The results are summarized in Table 2. Of the seven circular contigs of putative plasmids, the program identified four plasmids. The remaining three contigs (3 (3028 bp), 4 (5879 bp), and 7 (101,931 bp)) are presumed to represent three plasmids with novel types of replication origins.

As indicated by the findings of the PADLOC web server [26], the strain APEC 36 has been observed to possess a number of antiviral defense systems. The strain possesses a CRISPR/Cas9 type I-E system, a VSPR, a PDC, a PD-Lambda-1, an *ietAS*, a gabija, a Mokosh Type II, a restriction-modification type II, a DMS, and a *tmn* (see Supplementary Figure S1). The majority of these defense systems’ operons are located in the genome, and only the *tmn* is located on the plasmid (contig_7).

Table 3 provides a summary of the results of database searches for virulence factor genes as well as genes associated with antibiotic resistance [27]. This includes transporter genes [28,29], as well as drug targets [30]. Our results indicate that APEC 36 has several hundred genes associated with antibiotic resistance and virulence, which suggests a high pathogenic potential.

### 2.2. Genes and Potential Mechanisms of Antibiotic Resistance of E. coli Strain APEC 36

Subsequently, a comprehensive search for genes linked to antibiotic resistance mechanisms was conducted using the assembled genome of the APEC 36 strain and the RGI service in the CARD database [31]. The results of this search are presented in Table 4. The results indicate that the *E. coli* strain under study has the potential to reduce the efficacy of antibacterial drugs by inactivating the antibiotic, altering its target, replacing the target, expelling the antibiotic from the cell, and reducing the permeability of the cell wall. Transcriptional regulators and signal transduction systems may be involved in the activation of these cell-protecting processes.

Specifically, among the resistance genes identified by CARD RGI, more than half of the resistance genes function through the antibiotic efflux mechanism. These include genes that encode efflux pumps as well as regulators of their expression. Notably, all of these genes, with the exception *qacL*, are located on contig 5, which corresponds to the bacterial chromosome. In particular, the majority of the genes encode subunits of ABC transporters that are embedded in the inner membrane of the bacterial cell and belong to two major families: the major facilitator superfamily (MFS) and the resistance-nodulation-cell division (RND) family. Members of these families differ in structure and substrate specificity (MFS is somewhat broader), yet both can confer drug resistance in bacteria. Members of both families often form pores across both membranes and associate with outer membrane proteins such as TolC to form a tripartite complex [32,33].

Complexes that can be formed with members of the RND family include AcrAB-TolC, which protects against a wide range of drugs, including beta-lactams, tetracyclines, fluoroquinolones, rifamycin, phenicols, and so forth; AcrAD-TolC, which protects against beta-lactams and tetracyclines; and so on. In addition, the complexes can be formed with members of the RND family, including AcrAB-TolC (which protects against a wide range of drugs, including beta-lactams, tetracyclines, fluoroquinolones, rifamycin, phenicols, etc.), AcrAD-TolC (which protects against beta-lactams, tetracyclines, fluoroquinolones, rifamycin, phenicols, etc.), AcrEF-TolC (which moves beta-lactams and fluoroquinolones), MdtABC-TolC (which moves aminocoumarins), and MdtEF-TolC (which moves macrolides, fluoroquinolones, and penams). The MFS family includes the following complexes: ErmAB-TolC (which excretes fluoroquinolones), ErmKY-TolC (which excretes tetracyclines), MdtNOP-TolC (which excretes nucleoside antibiotics), and so forth [34,35,36]; and some MFS transporters, including Tet(A), which forms homodimers and acts against tetracyclines [37]. MdfA, which facilitates the cell’s clearance of tetracyclines and disinfectants, and CmlA5, which is anti-phenicol, operate in a TolC-independent manner [38]. Other groups include the KpnEF transporter of the small drug resistance (SMR) family, which eliminates macrolides, aminoglycosides, cephalosporins, tetracyclines, peptide antibiotics, rifampin, and other compounds [39].

Furthermore, the *E. coli* strain under investigation harbors the E448K mutation within the gene encoding GlpT. The mutation impairs the efficiency of fosfomycin antibiotic transport into the cell through the GlpT transporter [40,41]. Since fosfomycin inhibits the cytoplasm-localized MurA enzyme, which catalyzes the initial step of cell wall biosynthesis, namely the reaction of phosphoenolpyruvate addition to UDP-GlcNAc to form UDP-enoylpyruvyl-GlcNAc, the reduced antibiotic transport mitigates the adverse effects of fosfomycin.

With regard to the regulatory proteins involved in the control of antibiotic defense mechanisms, these include EmrR, H-NS, LeuO, RsmA, AcrS, CpxA, BaeR, CRP, GadX, KdpE, MarA, and the EvgA-EvgS system. ErmR is a member of the TetR family of proteins that functions as a negative regulator of the ErmAB operon [42]. AcrS and AcrR have been demonstrated to repress the *acrAB* operon [43,44], while MarA has been shown to activate the AcrAB-TolC efflux system [45]. The *marA* gene is a component of the *marRAB* operon, which is subject to negative regulation by the MarR repressor [45]. The Y137H and G103S mutations were identified in MarR, which is known to result in elevated expression of *marRAB* and *mdtE* [46] and, subsequently, the activation of the AcrAB efflux pump [47]. The LysR family transcription factor LeuO has been demonstrated to stimulate the transcription of operons encoding AcrEF, MdtNOP, and other transporters [48]. Conversely, H-NS is a universal silencer and an antagonist of LeuO [49]. EvgAS is a two-component system comprising EvgS, a sensory kinase that detects environmental stress, and EvgA, a response regulator that regulates the activity of numerous proteins [50]. The SoxRS systems is also a two-component system, with the SoxR protein containing [2Fe-2S] clusters. The latter are oxidized by reactive oxygen species and other oxidants that occur under conditions of cellular stress. This results in the activation of SoxR, which induces the expression of *soxS*. In turn, this increases the expression of transporter genes (such as *acrAB*), genes involved in DNA repair and oxidative stress control systems, and decreases the expression of genes encoding porins, such as OmpF, in order to reduce cell membrane permeability [51]. The sequenced genome revealed the presence of the A12S mutation in the SoxS protein, which has the potential to enhance the expression of the AcrAB efflux pump genes and result in diminished cell wall permeability due to reduced OmpF porin production. Low antibiotic input and activated drug efflux are hallmarks of multiple drug resistance phenotypes observed in *E. coli* clinical isolates [52].

It can be reasonably deduced that the upregulation or mutation of efflux pumps plays a significant role in the development of antibiotic and disinfectant resistance in the strain, both under conditions of stress and in the absence of such conditions.

Additionally, the genome of the strain contains multiple genes that confer resistance through the antibiotic target alteration mechanism. These are antibiotic targets that have undergone specific mutations, rendering them insensitive to inhibition. For example, the penicillin-binding protein PBP3, which is an alternative name for transpeptidase, has undergone mutations at residues D350N and S357N, rendering it insensitive to beta-lactam antibiotics [53]. An additional example is the translation elongation factor EF-Tu. Typically, GTP-bound EF-Tu forms a complex with aminoacyl-tRNA. Subsequently, the entire complex attaches to the ribosome, where EF-Tu hydrolyzes GTP and catalyzes the incorporation of the amino acid into the nascent polypeptide chain. Subsequently, the EF-Tu-GDP complex dissociates from the ribosome. With the assistance of another translation factor, EF-Ts, EF-Tu replaces GDP with GTP, thereby initiating another cycle. Elfamycin antibiotics, including pulvomycin and kirromycin, inhibit the activity of EF-Tu. They prevent the aminoacyl-tRNA binding step to the EF-Tu-GTP complex. The R234F mutation, which is present in two copies of the EF-Tu gene in our *E. coli* strain, should render it insensitive to these antibiotics [54,55].

The *E. coli* strain under investigation also harbors a number of genes that function by the mechanism of antibiotic inactivation. The genes encode enzymes that facilitate the degradation of the antibiotic or the addition of extra groups to it, which ultimately results in the loss of its activity. Of the 11 such genes that were identified, only four are located on the chromosome, and another four on contig 2, which corresponds to the plasmid, and another three on contig 8, which is also a plasmid. Subsequently, we will discuss the genes in relation to their role in antibiotic resistance mechanisms.

Beta-lactams were the first antibiotics to be discovered and remain the most diverse and widely used group of antibiotics. The mechanism of action of beta-lactams is the binding of the antibiotic to transpeptidase, an enzyme that catalyzes the formation of interpeptide cross-links in the cell wall. This binding occurs due to the molecular similarity of the antibiotic to the dimer of D-alanine, the natural substrate of transpeptidase. Subsequently, the beta-lactam ring is opened, resulting in the formation of a covalent bond with serine within the active site of transpeptidase, which inhibits the enzyme irreversibly. The most prevalent mechanism of resistance to beta-lactams is the production of beta-lactamases. These enzymes belong to the hydrolase class and function by hydrolyzing the amide bond in the beta-lactam ring, thereby rendering the antibiotic inactive. Among the enzymes whose genes were identified in the strain genome through sequencing, five beta-lactamases were detected that confer resistance to beta-lactam antibiotics by cleaving the beta-lactam ring. Two representatives of the TEM family, one enzyme of the CTX-M family (specifically CTX-M-169) and one of the OXA family (OXA-10 according to CARD RGI or OXA-48 according to DFAST), were identified. Additionally, an AmpC beta-lactamase was detected. As annotated by DFAST, the beta-lactamase was identified as CTX-M-169, one of the OXA family (specifically OXA-10 according to CARD RGI or OXA-48 according to DFAST), and one AmpC beta-lactamase. Both the TEM and CTX-M families are classified as class A beta-lactamases according to Ambler’s classification system. These enzymes are frequently classified as extended-spectrum beta-lactamases, which possess the ability to degrade not only penicillins but also cephalosporins and monobactams. It is fortunate that these enzymes are rarely resistant to inhibitors. The OXA family is classified as a class D beta-lactamase. These enzymes are frequently carbapenemases, and their sensitivity to inhibitors is variable. AmpC beta-lactamase belongs to Ambler’s class C, which is distinguished by variable substrate specificity and frequently observed insensitivity to inhibitors [56]. The remaining three enzymes conferred resistance to aminoglycosides, namely two ANT(3′) proteins (encoded by the *aadA* gene) and one AAC(3) protein (encoded by the AAC(3)-IId gene). AAC(3) utilizes acetyl-CoA to acetylate the 3-amino group of the antibiotic, whereas ANT(3′) transfers the adenyl group to the hydroxyl at the 3′ position in an ATP-dependent manner. Aminoglycoside antibiotics inhibit bacterial translation by binding to the 30S subunit of the ribosome. However, aminoglycosides modified by the enzymes identified in this study exhibit a markedly reduced affinity for the target [57,58,59]. Additionally, genes for three enzymes that similarly modify other groups of antibiotics were identified: chloramphenicol acetyltransferase (*catIII* gene), streptothricin acetyltransferase (*sat2* gene), and rifampin ADP-ribosyltransferase (*arr2* gene). Chloramphenicol targets protein synthesis on ribosomes (it binds to the 50S subunit), yet loses its activity after acetylation by the CAT enzyme [60]. Similarly, streptothricin binds to the 30S subunit of the ribosome. Following acetylation by the SAT enzyme, the interaction is no longer possible [61]. Rifampin, in turn, binds to the β-subunit of bacterial RNA polymerase, inhibiting transcription. The enzyme Arr, using NAD+ as a substrate, ADP-ribosylates rifampin, thereby removing the interference with RNA polymerase activity [62].

Some genes confer resistance through the mechanism of “antibiotic target replacement”, whereby they encode alternative enzymes that are not inhibited by the antibiotics. In the case of the strain under consideration, these are alternative enzymes in the folate biosynthesis pathway, which is required for the biosynthesis of purines (which in turn are required for DNA and RNA synthesis and cell proliferation) and a number of other metabolic pathways. The biosynthesis of tetrahydrofolate, which possesses reducing properties and is the active form of folate, in bacteria comprises three steps. Firstly, the enzyme dihydropteroate synthase (DHPS) catalyzes the formation of 7,8-dihydropterolate (DHP) from 6-hydroxymethyl-7,8-dihydropterin diphosphate (DHPP) and p-aminobenzoic acid (pABA). Subsequently, DHP is transformed into 7,8-dihydrofolate (DHF). In the third step, 7,8-dihydrofolate (DHF) is reduced to 5,6,7,8-tetrahydrofolate (THF). This final step is catalyzed by dihydrofolate reductase (DHFR). Sulfonamide antibiotics inhibit DHPS, which catalyzes the initial step of this pathway, and diaminopyrimidines act on DHFR, which is responsible for the final step. The *sul* genes (comprising two *sul2* and one *sul3* genes) encode alternative forms of DHPS that are insensitive to the action of sulfonamides. Additionally, *dfrA14* encodes DHFR that is not inhibited by diaminopyrimidine antibiotics. Accordingly, the genome sequence data indicated that the *sul* and *dfrA14* genes enable the APEC 36 *E. coli* strain to synthesize folate in response to both sulfonamides and diaminopyrimidines [63,64]. Furthermore, the *qnrS1* gene was identified, which is typically located on plasmids but was observed to be present on the chromosome. This gene confers resistance to fluoroquinolones through the mechanism of antibiotic target protection. As the target of quinolones is DNA gyrase, the action of these antibiotics results in the inability of the bacterial genome to replicate. The QnrS1 protein is capable of binding to the gyrase, thereby competing with the antibiotic. The interaction of QnrS1 with the enzyme does not interfere with the gyrase functioning and allows normal catalysis even in the presence of fluoroquinolones [65].

**Figure 2:**
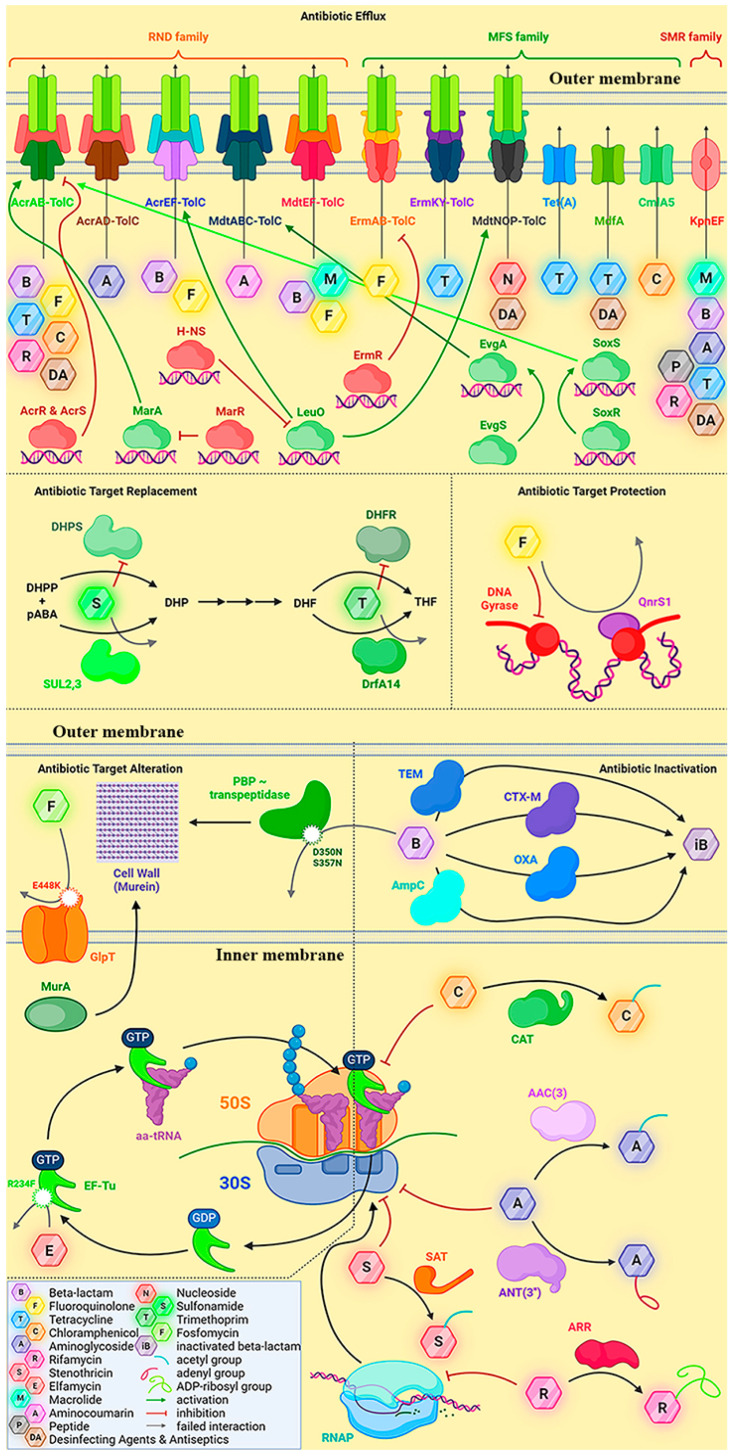
The antibiotic resistance mechanisms proposed for the *E. coli* APEC 36 strain, based on the analysis of its sequenced genome.

The drug resistance mechanisms identified for the *E. coli* APEC 36 strain are presented in Figure 2.

### 2.3. PCR Verification of the Presence of Virulence and Antibiotic Resistance Genes

Additionally, the presence of genes associated with virulence and antibiotic resistance was evaluated through analytical PCR (Table 5). The data indicated the presence of specific genes associated with the AREC pathotype, namely *hlyF* (a gene encoding for a virulence factor known as avian hemolysin) and *iutA* (a gene encoding for the receptor for the siderophore aerobactin). Among the genes of general pathogenicity, the presence of *kpsMT* and *iroN* genes was confirmed. Genes characteristic of the ExPEC group (UPEC) were represented by *upaG* (mediates adhesion to bladder epithelial cells) and *fimH* (encodes fimbrial adhesin). Furthermore, specific DEC/IPEC marker genes of the *estI*, *eltA*, *subA*, *eastI*, and *iha* group were identified. The presence of genes encoding beta-lactamases belonging to the CTX-M and TEM families was also confirmed by PCR.

### 2.4. Physiological Characteristics of E. coli Strain APEC 36

It is important to note that the presence of genes associated with pathogenicity and antibiotic resistance does not necessarily indicate the presence of the corresponding phenotype in the strain. This may be attributed to point mutations occurring in the genes themselves and in their regulatory regions or transcription factors. Subsequently, the antibiotic resistance of strain APEC 36 was evaluated to ascertain the functionality of the identified genes (Table 6). The results demonstrated that the strain exhibited resistance to several cephalosporins (cefuroxime, cefotaxime, and cefixime), fluoroquinolones (ciprofloxacin and levofloxacin), tetracyclines, sulfonamides, and chloramphenicol.

**Figure 3:**
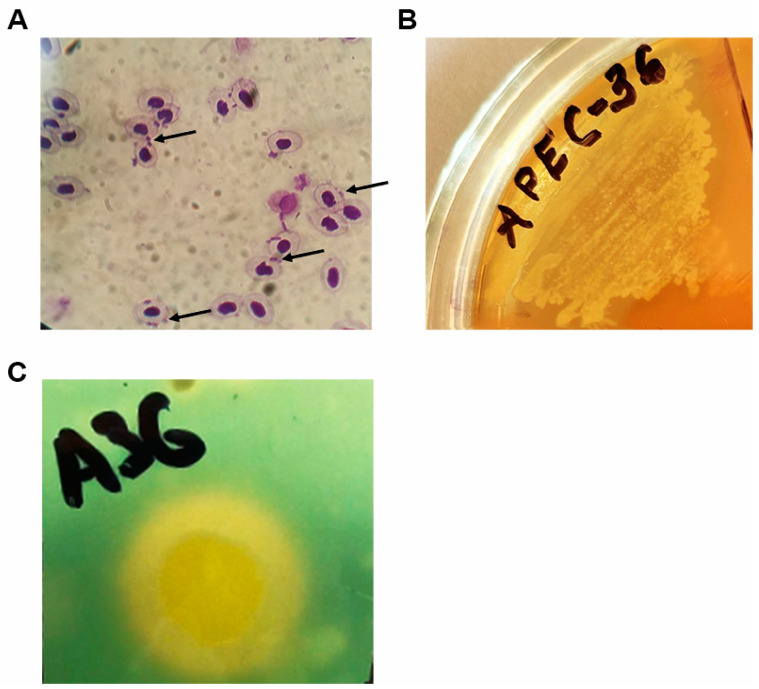
Assessment of pathogenicity of *E. coli* strain APEC 36. (**A**). An example of specific adhesion of *E. coli* APEC 36 on chicken red blood cells (arrow), 30 min, staining with gentian violet, 1000×. (**B**). An example of *E. coli* growth on chicken blood agar: viewed with incident light–obvious hemolysis. (**C**). Assessment of the siderophore production level.

Given that the strain in question exhibited the presence of both *iha* and *fimH* genes as determined by PCR (see Table 5), we proceeded to assess its capacity for adhesion to erythrocytes. APEC 36 exhibited minimal adhesion to human red blood cells (IAM = 1.47) and low adhesion to chicken red blood cells (IAM = 2.16) (Figure 3A). Furthermore, the strain was observed to lack hemolytic activity against human blood, yet exhibited the ability to lyse chicken red blood cells (Figure 3B). This phenotype aligns with the absence of the hlyA lysine gene and the presence of the hlyF lysine gene (Table 5).

The functionality of the iron uptake system was evaluated through the estimation of siderophore production levels. The coefficient was found to be 1.67 (Figure 3C), which lends support to the phenotypic manifestation of the *iroN* and *iutA* genes (Table 5).

**Figure 4:**
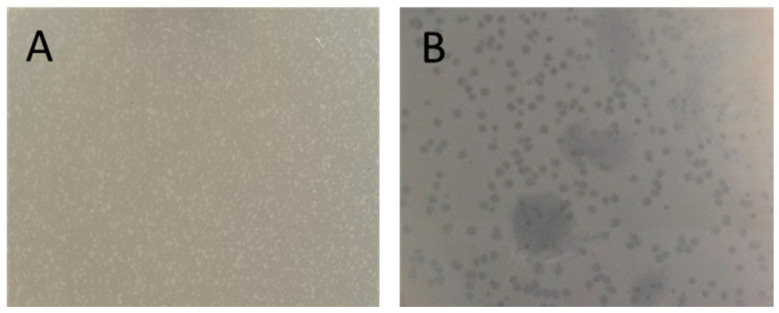
APEC 36 strain does not contain lysogenic prophages. (**A**) *E. coli* APEC 36; (**B**) control lysogenic prophage-carrying strain.

Subsequently, the strain was examined for the presence of bacteriophages. No lysogenic phages were identified (Figure 4). These findings indicate the functionality of anti-phage defense systems, such as the CRISPR/Cas IE system encoded in the genome of the APEC 36 strain.

## 3. Discussion

It is established that healthy farm animals can serve as a source of microorganisms with high virulence potential due to the presence of pathogenicity genes encoding adhesins, invasins, toxins, immune system resistance factors, and proteins of iron uptake systems. The initial characteristic of a strain’s virulence potential is typically regarded as its affiliation with a specific phylogenetic group. The available evidence indicates that APEC representatives are more frequently associated with phylogroup B1 or F [66,67,68], which encompasses a diverse range of pathogens with a multitude of virulence factors. The isolated *E. coli* APEC 36 strain was found to carry genes for toxins, adhesins, protectins, genes for UPEC-specific proteins, and iron uptake systems (Table 5). Based on this set of genetic characteristics, the strain was classified as a representative of phylogroup B1, since B1 is the predominant group among Shiga toxin-producing *Escherichia coli* (STEC/EHEC) [69]. APEC36 bears a wide range of virulence determinants and appeared to be close to O157:H7 strain; it seems to have a high pathogenic potential with respect to both poultry and human beings.

Genome analysis revealed the presence of a number of antiphage defense systems, including the CRISPR/Cas9 type I-E system, the VSPR, the PDC, the PD-Lambda-1, the *ietAS*, the Gabija, the Mokosh Type II, the restriction-modification Type II, the DMS, and the *tmn*. This raises the question of how plasmids survive the action of these systems.

Plasmids may circumvent the majority of these defensive mechanisms due to their lack of characteristics associated with phage infections. For example, plasmids may not undergo extensive replication or transcription, which precludes their ability to trigger the action of the Gabija defense system. This system is triggered when there is a depletion of NTP or dNTP pools [70]. The action of some other system, such as *ietAS* or Mokosh, is dependent on specific functional viral proteins, including DNA polymerase, ssDNA binding protein, helicase, terminase, and so forth [71]. Since plasmid replication is dependent on host enzymes, it can avoid the action of such defensive systems.

It seems reasonable to suggest that plasmids are more susceptible to defense systems that are targeted DNA, such as restriction-modification (R-M) systems or CRISPR/Cas systems. In recent work, it has been proposed that plasmids could evade the action of the R-M system in two ways, depending on the size of the plasmid [72]. In the case of relatively small plasmids, the primary mechanism of evasion involves sequence change. In contrast, in the case of relatively large plasmids, the primary mechanism of evasion involves the addition of genes that inactivate the R-M system. Additionally, plasmids can circumvent the action of the functional CRISPR/Cas IE system of *E. coli*. This is achieved initially via an enhanced replication rate that could be faster than the rate of CRISPR/Cas IE system action [73]. Subsequently, the plasmids accumulate escape mutations in targeted protospacers and become persistent in *E. coli* strains. It is plausible that plasmids may utilize multiple strategies to evade the defensive systems of *E. coli*.

The fight against such potentially dangerous strains is often complicated by their ability to survive under antibiotic pressure due to the presence of antibiotic resistance systems, which render the strains more resilient to the effects of antibiotics. This is a direct consequence of the active use of antibacterial drugs throughout the poultry production cycle in the majority of countries worldwide.

The most commonly utilized antibiotics for the prevention and treatment of bacterial infections in poultry within the industrial context are tetracyclines, fluoroquinolones, and sulfonamides. For this reason, a high incidence of strains resistant to these antibiotics has been documented for some time [74,75,76]. It is regrettable that the Food and Drug Administration (FDA) has approved the use of certain antibiotics in both animals and humans, including those classified as Category I antimicrobials, which are considered highly important for the treatment of severe human infections. Such drugs include, in particular, cephalosporins. For example, ceftiofur is employed for the control of omphalitis in broilers. It has been demonstrated that the proportion of *E. coli* strains exhibiting resistance to this drug was markedly elevated in chickens that received it as a prophylactic supplement [77]. Conversely, on farms where ceftiofur was discontinued, a reduction in the resistance of *E. coli* to this broad-spectrum cephalosporin was observed in healthy broilers [78].

It is of particular importance to note that resistance to specific antimicrobials can occur in the presence of both related and unrelated antimicrobials. For example, the resistance of *E. coli* to tetracycline in turkey flocks was found to be increased when not only tetracycline but also injectable aminoglycosides were used. Furthermore, a notable correlation between beta-lactam resistance and the administration of streptogramine in the diet has been documented [79].

According to data from the World Health Organization (WHO), the utilization of antibiotics in veterinary medicine is currently approximately twice the quantity of pharmaceuticals employed in human medicine. In light of these circumstances, efforts are being made to legally restrict the use of antibacterial drugs in agricultural practice with the aim of reducing the probability of the emergence and spread of antibiotic-resistant strains of microorganisms. In 2013, the FDA restricted the use of low-dose antibiotics as growth promoters in livestock, in accordance with the Guidance for Industry (GFI) #213. These antibiotics are important for human medicine [80]. Subsequently, in 2014, a comparable prohibition on select AGPs was enacted in Canada [81]. A number of countries within the Organisation for Economic Co-operation and Development (OECD) have implemented restrictions on the use of AGPs (e.g., Mexico, South Korea, New Zealand), whereas in other countries (e.g., Japan), the use of AGPs remains permitted [82]. The use of AGPs is not prohibited in the majority of non-OECD countries. These include major poultry producers such as China, Brazil, Russia, Argentina, India, Indonesia, the Philippines, and South Africa [82]. Furthermore, Regulation (EU) 2019/6 came into force in European countries on 28 January 2022. This regulation states that antimicrobial medicinal products may not be used for routine prophylaxis, defined as the administration of a medicinal product before the appearance of clinical signs of disease in order to prevent the occurrence of disease or infection. In Russia, the use of antibiotics in agriculture is also subject to regulation [83]. In particular, a list of feed antibiotics that are authorized for use has been established. However, for a variety of reasons, antibacterial drugs are often used in a haphazard manner or in violation of the prescription regime [84].

The *E. coli* strain APEC 36, as described in this work, contains a wide range of resistance genes to various antibacterial drugs within its genome (Table 4). A detailed examination of the genotype (Table 4) in conjunction with the identified phenotypic parameters of antibiotic resistance (Table 6) has enabled us to ascertain the genetic determinants, the products of which appear to directly contribute to the observed profile of reduced antibiotic sensitivity of the strain.

It is important to note that APEC 36 is sensitive to carbapenems and aminoglycosides (Table 6). Consequently, the genes whose products provide resistance to these drugs are nonfunctional under conditions studied. It is noteworthy that these genes encompass determinants of both specific and multidrug resistance. Consequently, AAC(3)-IId, AadA, ArcD, and KdpE are solely responsible for conferring resistance to aminoglycosides, while BaeR and CpxA are involved in this process for both aminoglycosides and aminocoumarin antibiotics. Additionally, KpnEF, MarA, SoxS, and TolC contribute to the development of resistance to a diverse range of antibacterial drugs.

It is important to note that the SoxS protein does not play a direct role in the cellular defense mechanism against the antibiotic. It functions as a transcriptional regulator, activated by the sensor protein SoxR in response to oxidative stress [85,86]. The activation of the SoxRS regulon results in the expression of genes that encode a variety of multidrug efflux pumps, which facilitate the removal of antibacterial drugs from the cell. It is established that mutations in the SoxR gene result in elevated levels of SoxS expression in the absence of stress. This, in turn, gives rise to the formation of multidrug resistance in clinical strains, thereby ensuring the perpetuation of this trait over multiple generations [87].

A comparable situation arises with the MarRAB operon, which encompasses the transcriptional regulator MarA. MarR binds to the marRAB promoter and functions as a negative regulator of gene expression [88]. In the presence of inducers, MarR binds these inducers and is then unable to interact with DNA [89]. In such a situation, MarA binds the marRAB promoter and acts as an activator of the system [90]. Therefore, if MarA fails to function for any reason, the entire operon becomes non-functional. Consequently, the corresponding proteins can also be excluded from the list of resistance determinants active in the cells of the strain under study.

The TolC protein constitutes a component of the major multidrug efflux pump of Gram-negative bacteria AcrAB-TolC. This pump forms a channel [91] that traverses through the periplasmic space and the outer membrane. The entrance to the channel through the inner membrane on the cytoplasmic side is determined by the AcrZ protein. The functionality of the AcrAB-TolC pump is contingent upon its complete assembly and the optimal operation of all its constituent components. Given that TolC does not afford protection against carbapenems, it would appear that this entire protein complex should be excluded from the list of resistance systems active in the APEC 36 strain. This assertion is corroborated by the observation that the strain under examination is susceptible to inhibitor-protected penicillins. Given that penicillin antibiotics can effectively inhibit the growth of APEC 36 in the presence of clavulanic acid, its resistance to this class of compounds is evidently determined by beta-lactamases rather than by multidrug efflux pumps. Consequently, we have a second line of evidence indicating the non-functionality of the AcrAB-TolC system.

In light of the other resistance determinants whose genes were identified in the genome of the strain under investigation through sequencing, it is notable that AcrR serves as a modulator of AcrAB-TolC pump gene expression [92]. Similarly, AcrS has been demonstrated to act as a repressor of the AcrAB efflux complex, and is associated with the expression of AcrEF. It is postulated that AcrS regulates a switch between AcrAB- and AcrEF-mediated efflux. Given that penicillin resistance has been demonstrated to be independent of efflux pumps (and thus the AcrEFS system, analogous to AcrAB-TolC, is non-functional), it can be concluded that acrR and acrEFS are not significant contributors to resistance.

In addition, the cAMP receptor protein (CRP) has been identified as a potential determinant of resistance for penicillins and fluoroquinolones (Table 6). CRP is one of seven global regulators in *E. coli*, influencing the expression of nearly 490 genes. These genes are involved in a number of processes, including the regulation of multidrug efflux pumps [93]. The other transcriptional regulator is GadX. This protein is responsible for the activation of the glutamate decarboxylation system, which serves as a defensive mechanism of *E. coli* against acid stress [94]. GadX has also been demonstrated to activate drug efflux pumps [95], though it is not directly involved in resistance, unlike CRP. In addition to the aforementioned transcription factors, other proteins indirectly involved in antibiotic resistance include the H-NS protein, which is a DNA-binding protein with a central role in gene regulation and nucleoid structuring [96], the RsmA protein, which is RNA-binding protein regulating a set of genes including those of the Type III secretion system [97], and the two-component signal-transduction system EvgAS, which regulates the EmrKY pump [98].

By shifting the focus to an analysis of the factors influencing the resistance of the APEC 36 strain to specific antibacterial drugs, and considering the spectrum of resistance systems that are either dysfunctional or indirectly related to the development of reduced antibiotic sensitivity, we can conclude the following.

It is evident that resistance to sulfonamides (cotrimoxazole) is a consequence of the activity of the *sul2* and *sul3* gene products, which act by the mechanism of antibiotic target replacement (Figure 2). This ensures the appearance of alternative forms of DHPS in the cell that are not inhibited by the antibacterial drug.

The resistance to amphenicol (chloramphenicol) appears to be due to the proteins CatIII (chloramphenicol acetyltransferase, which ensures inactivation of the antibiotic), CmlA5 (efflux pump), and MdtM (also an antibiotic efflux pump) [99]. Conversely, CatIII and CmlA5 are exclusively specific for amphenicols, while MdtM can also function with antibacterial drugs belonging to the fluoroquinolone group.

It has been proposed that insensitivity to tetracyclines may be related to the EmrKY, MdfA, and Tet(A) systems. All are efflux pumps, with MdfA being largely analogous to the amphenicol-specific CmlA.

With regard to fluoroquinolones, the most critical defense systems of the bacterial cell appear to be EmrRAB, MdtEFHM, and QnrS1. In this instance, MdtEF represents a multidrug efflux pump that may also exhibit activity against penicillins [100]. Given that the APEC 36 strain displays sensitivity to penicillins in the presence of clavulanic acid, it can be concluded that this system is ineffective under the conditions studied. In contrast, MdtM and MdtH may be functional. While the MdtH efflux pump is specific for fluoroquinolones [101], MdtM has broader substrate specificity [99] and may also confer APEC 36 immunity to amphenicols.

Another fluoroquinolone-specific drug efflux pump is EmrRAB [102], and QnrS1 is a specific protein that interacts with regions of gyrase besides the DNA-binding groove, potentially allowing more specific binding and destabilization of the topoisomerase-DNA-quinolone cleavage complex [103]. Therefore, while EmrRAB and MdtHM facilitate the extrusion of fluoroquinolones from the cytoplasm, QnrS1 operates in accordance with the mechanism of antibiotic target protection (Figure 2). It is noteworthy that *qnrS1* is typically encoded by plasmids [104]. However, in the APEC 36 strain, this gene is located in the genome, indicating that the determinants of antibiotic resistance may be captured in the genome and stably transmitted from generation to generation.

Upon returning to the subject of beta-lactam antibiotics, it can be stated with a high degree of confidence that the *E. coli* strain under study possesses at least one beta-lactamase enzyme capable of degrading penicillin antibiotics. However, this enzyme is inhibited by clavulanic acid. The APEC 36 genome encodes four distinct beta-lactamase variants. These are OXA, TEM, CTX-M, and AmpC. AmpC activity has been demonstrated to be independent of clavulanic acid presence [105]. This leads to the conclusion that AmpC is inactive. OXA beta-lactamases confer resistance to ampicillin and oxacillin, although some of them are capable of degrading cefotaxime, cefepime, and ceftazidime molecules. The beta-lactamase OXA-10 detected in the strain belongs to this category and should provide resistance to ceftazidime, cefepime, and aztreonam [105]. The results demonstrate that strain APEC 36 is susceptible to these antibiotics (Table 6), indicating that the beta-lactamase OXA-10, similar to AmpC, is nonfunctional. Therefore, in the context of penicillin antibiotics, the sole determinant of observed antibiotic resistance is the TEM-1 beta-lactamase. The aforementioned conclusion is indirectly corroborated by the previously published paper, wherein the genomic analysis of *E. coli* strains isolated from diseased chickens in the Czech Republic [106] demonstrated that the multiresistant phenotype observed in the majority of sequenced strains was predominantly based on resistance to β-lactams and quinolones, which were associated with TEM-type beta-lactamase genes and chromosomal gyrA mutations. It is important to note that, despite the existence of at least 19 distinct TEM inhibitor-resistant β-lactamases, the enzyme type identified in this study is clearly a clavulanic acid-sensitive variant.

The protection of the APEC 36 strain against cephalosporins also appears to be determined, at least in part, by the presence of TEM-1 beta-lactamase. An alternative enzyme that may contribute is the CTX-M-169 beta-lactamase. In general, CTX-beta-lactamases readily hydrolyze ceftazidime. However, our strain is sensitive to this drug, suggesting either nonfunctionality of CTX-M-169 (in which case, the resistance to cephalosporins depends entirely on TEM-1) or the inability of this enzyme variant (CTX-M-169) to degrade ceftazidime specifically.

It is noteworthy that the PBP3 protein is listed among the determinants of antibiotic resistance that can influence resistance to beta-lactams (Table 6). The protein is involved in cell wall synthesis and is targeted by beta-lactam antibiotics. Mutations in PBP3 can result in resistance to penicillins and cephalosporins through the mechanism of antibiotic target alteration [107]. However, in the presence of clavulanate, APEC 36 is sensitive to penicillins, indicating that PBP3 does not carry resistance mutations.

## 4. Materials and Methods

### 4.1. Isolation and Identification of E. coli Strain APEC 36

From 2016 to 2018, we conducted a post-mortem examination of the organs of broiler chickens (*Gallus gallus* L.) of the Ross 308 cross that had succumbed to generalized colibacillosis at a large poultry farm in the Perm region of Russia. The *E. coli* strain APEC 36 was isolated from the liver of a seven-day-old (December 2017) and subsequently deposited in the Ex Culture Collection of the Department of Biology, Faculty of Biotechnology, University of Ljubljana (Univerza v Ljubljani, Slovenia) under the designation L-5866.

The isolation and identification of the strain was conducted in accordance with the bacteriological method recommended by the Order on Unification of Microbiological Research Methods No. 535, 1985. The isolated strain was verified using the diagnostic test system ENTEROtest 16 (Erba Lachema s.r.o., Brno, Czech Republic), which is a Russified version of Microb-2 (Microbiological System Monitoring Microb-2, SMMM-2). The SMMM-2 was employed along with the PCR amplification of the 16S RNA fragment using primers 16s-F: GACCTCGGTTTAGTTCACAGA, 16s-R: CACACGCTGACGCTGACCA [108]. The resulting product was then subjected to Sanger sequencing.

### 4.2. Antibiotic Sensitivity Test

The determination of antibiotic sensitivity was conducted in accordance with the clinical guidelines “Determination of sensitivity of microorganisms to antimicrobial agents” of the Interregional Association of Clinical Microbiology and Antimicrobial Chemotherapy (MACMAX Version-2018-03). The sensitivity to antibiotics was determined for beta-lactams (ampicillin (10 µg), cefotaxime (5 µg), ceftazidime (10 µg)), as well as for aminoglycosides (amikacin (30 µg), gentamicin (10 µg)), tetracyclines (tetracycline (30 µg)), fluoroquinolones (ciprofloxacin (5 µg), levofloxacin (5 µg)), and sulfonamides (co-trimoxazole (trimethoprim/sulfamethoxazole, 1. The minimum inhibitory concentrations (MICs) of 25/23.75 µg were determined by the disk-diffusion method (MACMAX, version 2015-02, EUCAST, version 11.0, valid from 1 January 2021). The production of extended-spectrum beta-lactamases (ESBLs) was determined by the double-disc method.

### 4.3. The Hemolysis Test

The culture’s hemolytic ability was determined by sowing it on 5% blood agar using human or chicken erythrocytes. The presence of hemolysis was assessed visually by observing the formation of a clear zone around the streaks after 24 h of culture incubation at 37 °C.

### 4.4. Iron Uptake Test

The level of siderophore production was tested as previously described [109]. It was estimated semi-quantitatively by applying a coefficient defined as the ratio of the diameter (in mm) of the yellow-orange zone on the agar plate to the diameter of the bacterial colony.

### 4.5. Lysogenic Phage Detection

The presence of bacteriophages within cells was determined through the use of the ultraviolet radiation induction method. A liquid culture of the strain under study, with an optical density of 2.0 according to McFarland, was distributed across the surface of Petri dishes. Subsequently, the dishes were irradiated with a DB-30-1 arc bactericidal lamp with a power of 30 W (wavelength 253.7 nm, UV-C spectrum) at a distance of 1 m from the dishes for 70 s. Following this, the dishes were covered with lids and incubated for 1 h at 37 °C. The resulting suspension was mixed with a culture of the sensitive strain *E. coli* DH5α, added to melted 0.6% agar (46 °C), mixed and layered on pre-prepared dishes with agarized Luria Bertani (LB) medium. The dishes were then incubated for 24 h at 37 °C. The formation of lysis zones in the sensitive strain was documented. In parallel, dishes with a negative control, lacking any cultures, were prepared.

### 4.6. Bacterial Adhesion to Red Blood Cells

The study of bacterial-specific adhesion to red blood cells was conducted in accordance with the Brilis method in Eppendorf tubes [110]. To account for the adhesive properties of bacteria, human red blood cells (type O, Rh+) and chicken erythrocytes were utilized. The erythrocytes were washed in saline phosphate buffer (PBS), then diluted to 10^8^ cells/mL. The bacteria were cultivated overnight, rinsed with a phosphate buffer, and resuspended to a concentration of 10^8^ cells/mL. Subsequently, a bacterial suspension was combined with an erythrocyte mass in a 1:1 ratio, and incubated at 37 °C with agitation at 120 rpm for 30 min. Blood smears were prepared and stained with a 0.5% solution of gentian violet. In examining the preparations under optical microscopy, three key indicators were considered: the average adhesion index (AAI), which represents the mean number of microorganisms attached to the surface of a single red blood cell; the adhesion coefficient (AC), which denotes the percentage of red blood cells with bacteria on their surface; and the index of adhesiveness of microorganism (IAM), which is the ratio of AAI and AC. Counting was conducted on 100 cells, with the entire glass slide being examined. Based on the IAM values, the microorganisms were classified as follows: non-adhesive (IAM < 1.75), low-adhesive (IAM = 1.76–2.49), medium-adhesive (IAM = 2.50–3.99), and highly adhesive (IAM > 4.0).

### 4.7. Phylogenetic Group Determination

The phylogenetic group was determined using multiplex polymerase chain reaction (quadruplex PCR) with primers according to Clermont O. et al. (2013) [111]. The results were interpreted using the key for determining the phylogenetic group. As a control, *E. coli* strains B2, BJ30, BJ32, and BJ33 of phylogroup B2 from the collection of the Faculty of Biotechnology, University of Ljubljana (Univerza v Ljubljani, Slovenia) were used.

### 4.8. PCR Screening for Virulence and Beta-Lactamase Genes

The following gene fragments were amplified: those encoding toxins (*cnf1*, *east1*, *ehxA*, *estI*, *estII*, *eltA*, *hlyA*, *hlyF*, *stx1*, and *stx2*), adhesins (*fimH*, *papC*, *sfaDE*, *afa/draBC*, *iha* and *flu*), protectins (*ompT*, *kpsMTII*, and *iss*), iron uptake system proteins (*iroN* and *iutA*), UPEC-specific protein (usp), and beta-lactamase genes (*bla*_TEM_, *bla*_SHV_, *bla*_OXA_, and *bla*_CTX-M_) using the primers listed in Table 7. The reaction mixtures were prepared using reagents produced by Syntol LLC (Moscow, Russia). The reactions were conducted on a DNA Engine Dyad thermocycler (Bio-Rad, Foster City, CA, USA) under the conditions previously described in the relevant literature sources, which also provided the sequences of the oligonucleotides. PCR products were separated by electrophoresis in 1% agarose gel in the presence of ethidium bromide. The bands were then visualized using a Gel-Doc XR gel documentation system (Bio-Rad, Foster City, CA, USA).

### 4.9. Genomic DNA Isolation and Assessment of Its Quality and Quantity

Genomic DNA from *E. coli* strain APEC 36 was isolated from overnight culture grown in LB using GeneJET Genomic DNA purification kit (Thermo Fisher Scientific, Waltham, MA, USA) according to the manufacturer’s instructions. DNA quality was assessed by electrophoresis in 1% agarose gel and using a Nanodrop spectrophotometer (Thermo Fisher Scientific). The 260/280 nm absorbance ratio demonstrated a value of 1.96, 260/230 nm, and an absorbance ratio of 2.21. According to the manufacturer’s recommendations, DNA quality is considered good for nanopore sequencing if the A260/A280 ratio is 1.8–2.0 and A260/A230 ratio is 2.0–2.2. Precise DNA concentration was determined using a Qubit 3.0 fluorimeter (Invitrogen, Thermo Fisher Scientific, Waltham, MA, USA). Sample preparation for fluorimetry was performed using the Qudye dsDNA HS Assay kit (Lumiprobe RUS, Moscow, Russia). The measured DNA concentration of APEC 36 strain was 209.86 ng/μL.

### 4.10. DNA Library Preparation for Nanopore Sequencing

Genomic libraries were prepared from 400 ng of genome DNA using SQK-LSK109 ligation kits (Oxford Nanopore, Oxford, UK) and additional modules and enzymes NEBNext Ultra II End repair/dA-tailing Module (NEB, cat # E7546), NEBNext Quick Ligation Module (NEB, cat # E6056), NEB Blunt/TA Ligase Master Mix (NEB, cat # M0367), and NEBNext FFPE Repair Mix (NEB, M6630) according to the manufacturer’s protocol (Ligation sequencing gDNA Native Barcoding Kit 24 V14 SQK-NBD114. 24 NBE, Oxford Nanopore). The first step involved DNA repair and preparation of the ends for ligation of native adaptors, in which 1 μL of diluted control DNA (DCS) was added to the reaction mixture according to the manufacturer’s protocol. The second step was to ligate the native barcode. Adapters were ligated for 20 min at room temperature, followed by overnight at 40 °C. In the third step, adaptors were ligated for 60 min at room temperature. Reaction mixtures were stirred on a Hula mixer (Elmi, Riga, Latvia) to avoid fragmentation of long DNA strands. Purification at all steps from reaction impurities, unligated adapters, and short fragments was performed using AMPure XP magnetic particles (Beckman Coulter Brea, CA, USA), after which DNA was washed with freshly prepared 70% ethanol. The library was eluted with nuclease-free water. DNA concentration was measured using a fluorimeter.

### 4.11. DNA Sequencing Using Nanopore Technology

The sequencing cell of the SpotON FlowCell R10.4.1 was loaded with 20 femtomoles of a pre-prepared DNA library. At the outset of the sequencing process, the cell was loaded with 900 active pores. Sequencing was conducted on a MinION instrument (Oxford Nanopore, Oxford, UK). The Guppy basecaller 6.5.7 software was employed to convert raw data into pod5 format and generate fastq basecalls. Debarcoding of samples was accomplished using the aforementioned software in conjunction with the basecalling procedure (high accuracy model). All reads with quality Q < 8 were excluded from subsequent data analysis.

### 4.12. Genome Assembly and Annotation

The prior genome assembly reads were trimmed using the Chopper software (version 0.6.0) with a minimum read length of 500 and a minimum Phred average quality score of 13. A de novo genome assembly was conducted using the Flye program (version 2.9.2.) in ONT regular reads mode with two rounds of polishing [127]. The quality of the assembly was evaluated using the programs Quast (version 5.2.0, [16]) and BUSCO (version 5.5.0, [17]). Genome annotation and strain identification were conducted using Prokka (version 1.14.6, [19]) with the default settings. The BV-BRC database services [22], including RASTtk [20], the Resistance Gene Identifier (RGI, version 6.0.3) service of the Comprehensive Antibiotic Resistance Database (CARD, version 3.2.9) [31], the PubMLST database [21], and the MiGA online server [18] were also utilized.

### 4.13. Phylogenetic Analysis

A phylogenetic analysis of the *E. coli* APEC 36 strain was conducted using the comprehensive genome analysis algorithm, accessible via the BV-BCR website [22]. It comprised a number of distinct phases. First, a set of reference and representative genomes that were most similar to the query genome were identified by Mash/MinHash [128], using a pre-formed database provided by PATRIC. Secondly, PGFams [129] were selected from all the aforementioned genomes, including the query genome, and aligned using MUSCLE [130]. Subsequently, the nucleotides from the genomes were mapped to this alignment. Ultimately, the entirety of the data was consolidated into a data matrix, which was then subjected to analysis by RaxML [131]. The tree’s support values were generated with the assistance of fast bootstrapping [132].

## 5. Conclusions

Summarizing the information obtained by sequencing the genome of strain APEC 36 and evaluating its phenotypic properties, it can be noted that the described *E. coli* strain exhibits a high degree of pathogenic potential. The genome contains genes for toxins and protectins, as well as functional adhesins, hemolysins, and proteins involved in iron uptake. The presence of multiple determinants of antibiotic resistance establishes the strain’s insensitivity to a wide range of antibacterial drugs. Conversely, the potential for antibiotic resistance remains unfulfilled, with numerous resistance determinants remaining unexpressed phenotypically. Notably, this is the case with the multidrug efflux systems AcrAB-TolC and MdtEF, as well as the beta-lactamases AmpC and OXA.

This correlation of phenotypic and genotypic traits suggests a probable history of the strain’s emergence. It is plausible that the strain was formed in an environment with some level of antibacterial drug pressure, which resulted in the acquisition of the corresponding resistance genes. However, as the influence of antibiotics waned and the corresponding resistance determinants were “canned,” the cell “turned them off” to enhance fitness by redirecting resources toward growth and reproduction. Consequently, the resistance genes are present within the cells, yet they are not functional. This fact serves to increase the clinical danger of the APEC 36 isolate. It would appear that relatively short-term exposure to moderate doses of antibiotics can result in the activation of previously dormant resistance determinants, rendering the cells sensitive to a significantly reduced spectrum of antibacterial drugs and complicating the treatment of infection. This prospect is particularly concerning given the inactivity of multidrug efflux pumps, including the AcrAB-TolC system, which is the primary one for Gram-negative bacteria.

In light of the considerable diversity of biological characteristics, virulence factors, and determinants of antibiotic resistance observed among strains of agricultural origin, a comprehensive study of these factors is of paramount importance for the effective control of epidemic and epizootic situations in agricultural enterprises and the prevention of the environmental dissemination of potentially pathogenic microorganisms. A comprehensive understanding of the biology (resistance determinants, transmissible potential) of antibiotic resistance development in the main representatives of the microbiocenosis of farm animals and poultry is essential for the effective development of measures and technologies to combat antimicrobial resistance.

## Figures and Tables

**Table 1:** Results of MLST analysis of *E. coli* strain APEC 36.

Gene	*adk*	*fumC*	*gyrB*	*icd*	*mdh*	*purA*	*recA*
**Allele**	10	11	4	12	8	18	2

**Table 2:** Plasmids identified in *E. coli* strain APEC 36 using PlasmidFinder 2.1.

Plasmid	Identity	Contig	Contig Length, bp	Position in Contig
Col(MG828)	95.79	Contig 1	1209	434…694
IncX1	98.4	Contig 2	54,843	23,254…23,627
p0111	98.64	Contig 6	75,049	29,044…29,928
IncI1-I(Alpha)	99.3	Contig 8	108,625	70,271…70,412

**Table 3:** General statistics on the number of genes characterizing the pathogenic potential of strain APEC 36.

Functional Group of Genes	Database	Number of Genes
	Victors	2
Associated with antibiotic resistance	CARD	95
	NDARO	21
	PATRIC	71
Encoding drug targets	DrugBank	383
	TTD	59
Transporter genes	TCDB	889
Virulence factor genes	PATRIC_VF	203
	VFDB	48
	Victors	212

**Table 4:** Genes associated with antibiotic resistance found in the genome of APEC 36 strain.

Gene (ARO Accession Number of the Reference Gene)	Gene Family	Class of Antibiotics	Mechanism of Resistance	Match with Reference Gene %
*aac(3)-IId* (3004623)	AAC(3)	Aminoglycosides	Antibiotic inactivation	100
*aadA* (3002602)	ANT(3″)	Aminoglycosides	Antibiotic inactivation	99.62
*acrA* (3000207)	RND (resistance-nodulation-cell division) antibiotic efflux pumps	Fluoroquinolones, cephalosporins, glycylcycline antibiotics, penicillins, tetracyclines, rifampicin, amphenicols, disinfecting compounds and antiseptics	Antibiotic efflux	100
*acrB* (3000216)	RND antibiotic efflux pumps	Fluoroquinolones, cephalosporins, glycylcycline antibiotics, penicillins, tetracyclines, rifampicin, amphenicols, disinfecting compounds and antiseptics	Antibiotic efflux	100
*acrD* (3000491)	RND antibiotic efflux pumps	Aminoglycosides	Antibiotic efflux	99.9
*acrE* (3000499)	RND antibiotic efflux pumps	Fluoroquinolones, cephalosporins, cefamycin, penicillins	Antibiotic efflux	100
*acrF* (3000502)	RND antibiotic efflux pumps	Fluoroquinolones, cephalosporins, cefamycin, penicillins	Antibiotic efflux	99.71
*acrS* (3000656)	Transcriptional regulator of RND antibiotic efflux pumps	Fluoroquinolones, cephalosporins, glycylcycline antibiotics, cefamycin, penicillins, tetracyclines, rifampicin, amphenicols, disinfecting compounds and antiseptics	Antibiotic efflux	100
*ampC* (3004290)	AmpC-type β-lactamase	Cephalosporins, penicillins	Antibiotic inactivation	98.14
*arnT* (3005053)	Phosphoethanolamine transferase	Peptide antibiotics	Target modification	63.52
*arr-2* (3002847)	Rifampin ADP-ribosyltransferase	Rifampicin	Antibiotic inactivation	100
*bacA* (3002986)	Proteins that are associated with undecaprenyl pyrophosphate	Peptide antibiotics	Target modification	100
*baeR* (3000828)	Transcriptional regulator of RND antibiotic efflux pumps	Aminoglycosides, aminocoumarin antibiotics	Antibiotic efflux	96.23
*catIII* (3002685)	Chloramphenicol acetyltransferase	Amphenicols	Antibiotic inactivation	99.53
*cmlA5* (3002695)	MFS (major facilitator superfamily) antibiotic efflux pumps	Amphenicols	Antibiotic efflux	100
*cpxA* (3000830)	Transcriptional regulator of RND antibiotic efflux pumps	Aminoglycosides, aminocoumarin antibiotics	Antibiotic efflux	100
*crp (* *3000518)*	Encodes CRP transcriptional regulator of RND antibiotic efflux pumps	Macrolides, fluoroquinolones, penicillins	Antibiotic efflux	99.52
*bla_CTX-M-169_* (3005592)	CTX-M β-lactamase	Cephalosporins	Antibiotic inactivation	100
*dfrA14* (3002859)	Trimethoprim-resistant dihydrofolate reductase	Diaminopyrimidines	Target substitution	100
*tufA* (3003369)	Encodes EF-Tu resistant to elphamycin	Elphamycins	Target modification	99.75
*emrA* (3000027)	MFS antibiotic efflux pumps	Fluoroquinolones	Antibiotic efflux	99.74
*emrB* (3000074)	MFS antibiotic efflux pumps	Fluoroquinolones	Antibiotic efflux	100
*emrK (3000206)*	MFS antibiotic efflux pumps	Tetracyclines	Antibiotic efflux	100
*emrR* (3000516)	MFS antibiotic efflux pumps	Fluoroquinolones	Antibiotic efflux	100
*emrY* (3000254)	MFS antibiotic efflux pumps	Tetracyclines	Antibiotic efflux	99.41
*eptA* (3003576)	Phosphoethanolamine transferase	Peptide antibiotics	Target modification	99.82
*evgA* (3000832)	Transcriptional regulator of MFS antibiotic efflux pumps, RND antibiotic efflux pumps	Macrolides, fluoroquinolones, penicillins, tetracyclines	Antibiotic efflux	100
*evgS* (3000833)	Transcriptional regulator of MFS antibiotic efflux pumps, RND antibiotic efflux pumps	Macrolides, fluoroquinolones, penicillins, tetracyclines	Antibiotic efflux	99.58
*gadX* (3000508)	Transcriptional regulator of RND antibiotic efflux pumps	Macrolides, fluoroquinolones, penicillins	Antibiotic efflux	100
*glpT* (3003889)	GlpT resistant to antibiotics	Phosphonic acid derivatives	Target modification	99.78
*hns* (3000676)	Encodes H-NS transcriptional regulator of MFS and RND antibiotic efflux pumps	Macrolides, fluoroquinolones, cephalosporins, cefamycin, penicillins, tetracyclines	Antibiotic efflux	100
*kdpE* (3003841)	Transcriptional regulator of *kdpDE* two-component regulatory system	Aminoglycosides	Antibiotic efflux	100
*kpnE* (3004580)	SMR (small multidrug resistance) antibiotic efflux pumps	Macrolides, aminoglycosides, cephalosporins, tetracyclines, peptide antibiotics, rifampicin, disinfecting compounds and antiseptics	Antibiotic efflux	82.2
*kpnF* (3004583)	SMR antibiotic efflux pumps	Macrolides, aminoglycosides, cephalosporins, tetracyclines, peptide antibiotics, rifampicin, disinfecting compounds and antiseptics	Antibiotic efflux	84.4
*leuO* (3003843)	Transcriptional regulator of MFS antibiotic efflux pumps	Nucleoside antibiotics, disinfecting compounds and antiseptics	Antibiotic efflux	99.04
*marA* (3000263)	Transcriptional regulator of RND antibiotic efflux pumps and porin with reduced permeability to β-lactams	Fluoroquinolones, monobactams, carbapenems, cephalosporins, glycylcycline antibiotics, cefamycin, penicillins, tetracyclines, rifampicin, amphenicols, penems, disinfecting compounds and antiseptics	Antibiotic efflux, cell wall permeability reduction	100
*mdfA* (3001328)	MFS antibiotic efflux pumps	Tetracyclines, disinfecting compounds and antiseptics	Antibiotic efflux	97.07
*mdtA* (3000792)	RND antibiotic efflux pumps	Aminocoumarin antibiotics	Antibiotic efflux	99.04
*mdtB* (3000793)	RND antibiotic efflux pumps	Aminocoumarin antibiotics	Antibiotic efflux	99.81
*mdtC* (3000794)	RND antibiotic efflux pumps	Aminocoumarin antibiotics	Antibiotic efflux	99.9
*mdtE* (3000795)	RND antibiotic efflux pumps	Macrolides, fluoroquinolones, penicillins	Antibiotic efflux	100
*mdtF* (3000796)	RND antibiotic efflux pumps	Macrolides, fluoroquinolones, penicillins	Antibiotic efflux	99.9
*mdtG* (3001329)	MFS antibiotic efflux pumps	Phosphonic acid derivatives	Antibiotic efflux	100
*mdtH* (3001216)	MFS antibiotic efflux pumps	Fluoroquinolones	Antibiotic efflux	99.75
*mdtM* (3001214)	MFS antibiotic efflux pumps	Fluoroquinolones, lincosamide antibiotics, nucleoside antibiotics, amphenicols, disinfecting compounds and antiseptics	Antibiotic efflux	98.01
*mdtN* (3003548)	MFS antibiotic efflux pumps	Nucleoside antibiotics, disinfecting compounds and antiseptics	Antibiotic efflux	99.71
*mdtO* (3003549)	MFS antibiotic efflux pumps	Nucleoside antibiotics, disinfecting compounds and antiseptics	Antibiotic efflux	100
*mdtP* (3003550)	MFS antibiotic efflux pumps	Nucleoside antibiotics, disinfecting compounds and antiseptics	Antibiotic efflux	98.36
*msbA* (3003950)	ABC (ATP-binding cassette) antibiotic efflux pumps	Nitroimidazoles	Antibiotic efflux	100
*bla_OXA-10_ (* *3001405)*	OXA β-lactamase, similar to OXA-10	Cephalosporins, penicillins	Antibiotic inactivation	100
*ftsI (pbpB)* (3004446)	Penicillin-binding proteins conferring resistance to β-lactam antibiotics	Cephalosporins, cefamycin, penicillins	Target modification	53.11
*pmrF* (3003578)	Phosphoethanolamine transferase	Peptide antibiotics	Target modification	100
*qacL* (3005098)	SMR antibiotic efflux pumps	Disinfecting compounds and antiseptics	Antibiotic efflux	93.64
*qnrS1* (3002790)	Quinolone resistance proteins	Fluoroquinolones	Protection of the antibiotic target	100
*rsmA* (3005069)	RND antibiotic efflux pumps	Fluoroquinolones, diaminopyrimidines, amphenicols	Antibiotic efflux	85.25
*sat2* (3002895)	Streptothricin acetyltransferase	Nucleoside antibiotics	Antibiotic inactivation	100
*soxR* (3000836)	Transcriptional regulator of ABC, MFS, and RND antibiotic efflux pumps	Fluoroquinolones, cephalosporins, glycylcycline antibiotics, penicillins, tetracyclines, rifampicin, amphenicols, disinfecting compounds and antiseptics	Target modification, antibiotic efflux	100
*soxS* (3000837)	Transcriptional regulator of ABC, MFS, and RND antibiotic efflux pumps and porin with reduced permeability to β-lactams	Fluoroquinolones, monobactams, carbapenems, cephalosporins, glycylcycline antibiotics, cefamycin, penicillins, tetracyclines, rifampicin, amphenicols, penems, disinfecting compounds and antiseptics	Target modification, antibiotic efflux, cell wall permeability reduction	100
*sul2* (3000412)	Sulfonamide resistance	Sulfonamides	Target substitution	100
*sul3* (3000413)	Sulfonamide resistance	Sulfonamides	Target substitution	100
*bla_TEM-1_* (3000873)	TEM β-lactamase	Monobactams, cephalosporins, penicillins, penems	Antibiotic inactivation	100
*tet(A)* (3000165)	MFS antibiotic efflux pumps	Tetracyclines	Antibiotic efflux	99.74
*tolC* (3000237)	ABC antibiotic efflux pumps, MFS antibiotic efflux pumps, RND antibiotic efflux pumps	Macrolides, fluoroquinolones, aminoglycosides, carbapenems, cephalosporins, glycylcycline antibiotics, cefamycin, penicillins, tetracyclines, peptide antibiotics, aminocoumarin antibiotics, rifampicin, amphenicols, penems, disinfecting compounds and antiseptics	Antibiotic efflux	100
*vanG* (3002909)	Glycopeptide resistance gene cluster, ligase Van	Glycopeptide antibiotics	Target modification	38.23
*yojI* (3003952)	ABC antibiotic efflux pumps	Peptide antibiotics	Antibiotic efflux	99.82

**Table 5:** Detection of virulence and antibiotic resistance genes by PCR.

Functional Group of Genes	Gene	Detected by PCR
Toxins	*east1*	+
	*ehxA*	−
	*eltA*	+
	*estI*	+
	*estII*	−
	*cnf1*	−
	*hlyA*	−
	*hlyF*	+
	*stx1*	−
	*stx2*	−
Adhesins	*afa/draBC*	*−*
	*iha*	*+*
	*fimH*	*+*
	*flu*	*−*
	*papC*	*−*
	*sfaDE*	*−*
Protectins	*iss*	−
	*kpsMTII*	+
	*ompT*	−
Iron uptake system	*iroN*	+
	*iutA*	+
UPEC-specific proteins	*usp*	−
	*upaG*	+
Beta-lactamases	*bla_CTX-M_*	+
	*bla_OXA_*	+
	*bla_SHV_*	−
	*bla_TEM_*	+

**Table 6:** Antibiotic resistance profile of *E. coli* strain APEC 36.

Antibiotic Group	Antibiotic	Growth Inhibition Zone Diameter (mm)	Sensitivity (S)/Resistance (R)	Putative Genome-Encoded Determinants of Resistance
Penicillins	Ampicillin	<14	R	AcrAB-TolC, AcrR, AcrEFS, AmpC, CRP, EvgAS, GadX, H-NS, MarRA, MdtEF, OXA-10, PBP3, SoxRS, TEM-1
	Amoxicillin/clavulanic acid	<19	S	
Cephalosporins	Cefuroxime	<19	R	AcrAB-TolC, AcrR, AcrEFS, AmpC, CTX-M-169, H-NS, KpnEF, MarRA, OXA-10, PBP3, SoxRS, TEM-1
	Cefotaxime	<17	R	
	Cefixime	<17	R	
	Ceftazidime	<19	S	
	Cefepime	<24	S	
Carbapenems	Meropenem	<15	S	MarA, SoxS, TolC
Aminoglycosides	Gentamicin	<14	S	AAC(3)-IId, AadA, ArcD, BaeR, CpxA, KdpE, KpnEF, TolC
	Amikacin	<15	S	
Fluoroquinolones	Ciprofloxacin	<24	R	AcrAB-TolC, AcrR, AcrEFS, CRP, EmrRAB, EvgAS, GadX, H-NS, MarRA, MdtEFHM, QnrS1, RsmA, SoxRS
	Levofloxacin	<19	R	
Tetracyclines	Tetracycline	<12	R	AcrAB-TolC, AcrR, AcrS, MarRA, EmrKY, EvgAS, H-NS, KpnEF, MdfA, SoxRS, Tet(A)
Trimethoprim/ sulfonamides	Cotrimoxazole	<11	R	Sul2, Sul3
Amphenicols	Chloramphenicol	<17	R	AcrAB-TolC, AcrR, MarRA, AcrS, CatIII, CmlA5, MdtM, RsmA, SoxRS

**Table 7:** Oligonucleotides used in the work.

Gene	Oligonucleotide Name and Sequences (5′->3′)	Amplicon Size, bp	Source
*east1*	east 11a: CCATCAACACAGTATATCCGA east 11b: GGTCGCGAGTGACGGCTTTGT	111	[112]
*ehxA*	ehxA-F: GCATCATCAAGCGTACGTTCC ehxA-R: AATGAGCCAAGCTGGTTAAGCT	534	[112]
*eltA*	LTA-1: GGCGACAGATTATACCGTGC LTA-2: CCGAATTCTGTTATATATGTC	696	[112]
*estI*	STa1: TCTTTCCCCTCTTTTAGTCAG STa2: ACAGGCAGGATTACAACAAAG	166	[112]
*estII*	STb-1: ATCGCATTTCTTCTTGCATC STb-2: GGGCGCCAAAGCATGCTCC	172	[112]
*cnf1*	CNF1-1: CTGACTTGCCGTGGTTTAGTCGG CNF1-2: TACACTATTGACATGCTGCCCGGA	1295	[113]
*hlyA*	hlyA1: GTCTGCAAAGCAATCCGCTGCAAATAAA hlyA2: CTGTGTCCACGAGTTGGTTGATTAG	561	[114]
*hlyF*	hlyF1: TCGTTTAGGGTGCTTACCTTCAAC hlyF2: TTTGGCGGTTTAGGCATTCC	444	[115]
*stx1*	stx1-F: ATAAATCGCCTATCGTTGACTAC stx1-R: AGAACGCCCACTGAGATCATC	180	[112]
*stx2*	stx2-F: GGCACTGTCTGAAACTGCTCC stx2-R: TCGCCAGTTATCTGACATTCTG	255	[112]
*afa/draBC*	afa/draBC-F: GGCAGAGGGCCGGCAACAGGC afa/draBC-R: CCCGTAACGCGCCAGCATCTC	592	[116]
*iha*	IHA-F: CTGGCGGAGGCTCTGAGATCA IHA-R: TCCTTAAGCTCCCGCGGCTGA	827	[112]
*fimH*	FimH1: CAGCGATGATTTCCAGTTTGTGTG FimH2: TGCGTACCAGCATTAGCAATGTCC	461	[117]
*flu*	flu-F: GGGTAAAGCTGATAATGTCG flu-R: GTTGCTGACAGTGAGTGTGC	508	[118]
*papC*	Pap1: GACGGCTGTACTGCAGGGTGTGGCG Pap2: ATATCCTTTCTGCAGGGATGCAATA	328	[119]
*sfaDE*	SFA-1: CTCCGGAGAACTGGGTGCATCTTAC SFA-2: CGGAGGAGTAATTACAAACCTGGCA	410	[119]
*iss*	iss-F: CAGCAACCCGAACCACTTGATG iss-R: AGCATTGCCAGAGCGGCAGAA	323	[112]
*kpsMTII*	kpsMT_II f: GCGCATTTGCTGATACTGTTG kpsMT_II r: CATCCAGACGATAAGCATGAGCA	270	[116]
*ompT*	ompT-F: TCATCCCGGAAGCCTCCCTCACTACTAT ompT-R: TAGCGTTTGCTGCACTGGCTTCTGATAC	496	[120]
*iroN*	iroN f: AAGTCAAAGCAGGGGTTGCCCG iroN r: GACGCCGACATTAAGACGCAG	925	[116]
*iutA*	iutA-F: GGCTGGACATCATGGGAACTGG iutA-R: CGTCGGGAACGGGTAGAATCG	301	[121]
*usp*	USP-N6: ATGCTACTGTTTCCGGGTAGTGTGT USP-N7: CATCATGTAGTCGGGGCGTAACAAT	1017	[122]
*upaG*	upaG-F: GATAGGCAAGGACGCAAGA upaG-R: GGTCGCAATATCCGTAGT	1218	[123]
*bla_CTX-M_*	CTX-M-A: CGCTTTGCGATGTGCAG CTX-M-B: ACCGCGATATCGTTGGT	551	[124]
*bla_OXA_*	OXA-10-F: GGCGAAAAAGTAAACCTC OXA-10-R: CAGTAAATCCTGCGCTAC	568	[125]
*bla_SHV_*	SHV-F: AGGATTGACTGCCTTTTTG SHV-R: ATTTGCTGATTTCGCTCG	392	[126]
*bla_TEM_*	TEM-C: ATCAGCAATAAACCAGC TEM-H: CCCCGAAGAACGTTTTC	516	[126]

## Data Availability

All data generated during and/or analyzed during the current study are available from the corresponding author on reasonable request.

## Supplementary Materials

The following supporting information can be downloaded at: https://www.mdpi.com/article/10.3390/antibiotics13100945/s1, Figure S1: Antiphage defensive systems of the *E. coli* APEC 36 strain.
