# Supplementary figures and images for: Determinants of Antibiotic Resistance and Virulence Factors in the Genome of Escherichia coli APEC 36 Strain Isolated from a Broiler Chicken with Generalized Colibacillosis
Source: Antibiotics (Basel). 2024 Oct 9;13(10):945. doi: 10.3390/antibiotics13100945 (PMC11504656; doi:10.3390/antibiotics13100945)

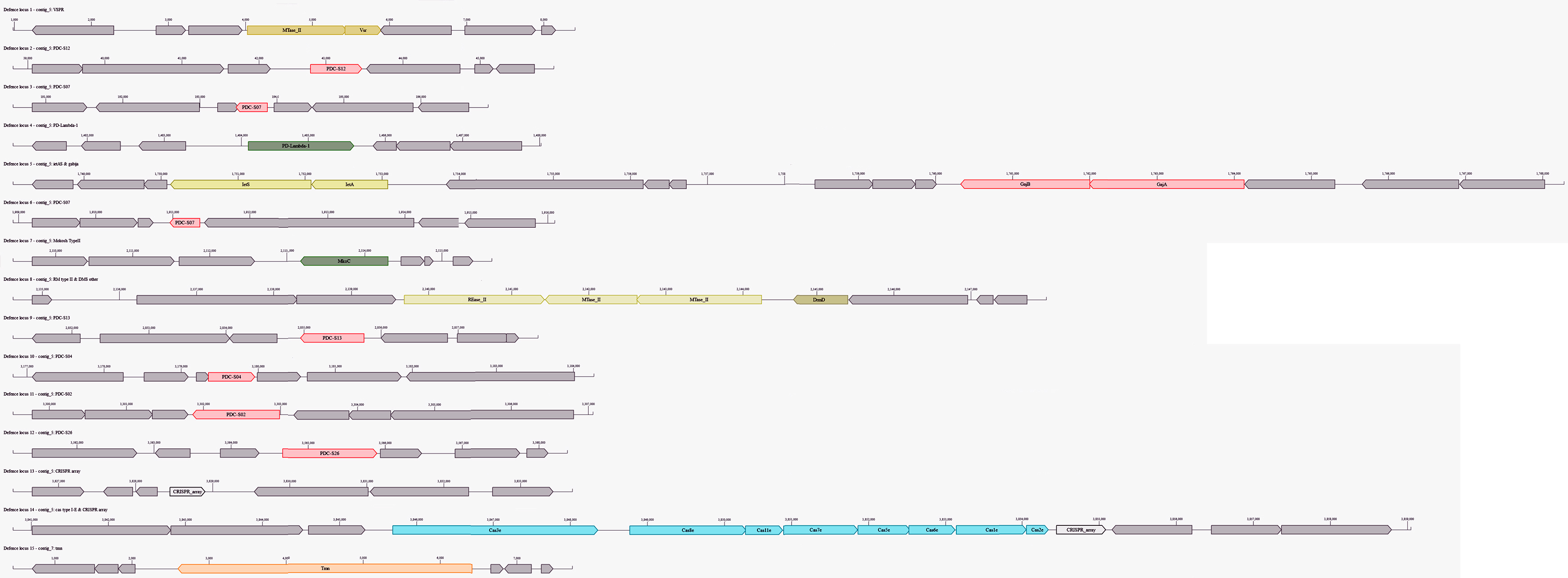

Supplement: Supplementary file 1 [file antibiotics-13-00945-s001.zip › antibiotics-3239251-supplementary/antibiotics-3239251-Figure S1.jpg]
